# MOIRAI: a compact workflow system for CAGE analysis

**DOI:** 10.1186/1471-2105-15-144

**Published:** 2014-05-16

**Authors:** Akira Hasegawa, Carsten Daub, Piero Carninci, Yoshihide Hayashizaki, Timo Lassmann

**Affiliations:** 1 RIKEN Center for Life Science Technologies (CLST), Riken Yokohama Institute, 1-7-22 Suehiro-cho, Tsurumi-ku, Yokohama, 230-0045 Kanagawa, Japan; 2 Preventive Medicine and Diagnosis Innovation Program (PMI), Riken Yokohama Institute, 1-7-22 Suehiro-cho, Tsurumi-ku, Yokohama, 230-0045 Kanagawa, Japan; 3 Department of Biosciences and Nutrition, Karolinska Institutet, SE-171 77 Stockholm, Sweden

**Keywords:** CAGE, Pipeline, Next generation sequencing

## Abstract

**Background:**

Cap analysis of gene expression (CAGE) is a sequencing based technology to capture the 5’ ends of RNAs in a biological sample. After mapping, a CAGE peak on the genome indicates the position of an active transcriptional start site (TSS) and the number of reads correspond to its expression level. CAGE is prominently used in both the FANTOM and ENCODE project but presently there is no software package to perform the essential data processing steps.

**Results:**

Here we describe MOIRAI, a compact yet flexible workflow system designed to carry out the main steps in data processing and analysis of CAGE data. MOIRAI has a graphical interface allowing wet-lab researchers to create, modify and run analysis workflows. Embedded within the workflows are graphical quality control indicators allowing users assess data quality and to quickly spot potential problems. We will describe three main workflows allowing users to map, annotate and perform an expression analysis over multiple samples.

**Conclusions:**

Due to the many built in quality control features MOIRAI is especially suitable to support the development of new sequencing based protocols.

**Availiability:**

The MOIRAI source code is freely available at http://sourceforge.net/projects/moirai/.

## Background

The next generation sequencing has taken a central role in genomics and transcriptomics. In recent years both the amount of data generated per sample and the number of samples used in a particular study has increased dramatically. Robust data processing workflows and a data management system are essential to deal with the flood of data.

Among assays to study the transcriptome cap analysis of gene expression (CAGE) technology specializes on detecting the 5’ end of transcripts [1,2]. RNAs can be quantified by counting the number of reads at a particular transcriptional start site (TSS). CAGE profiling has revealed an unexpected transcriptional complexity in both human and mouse [3]. More recently CAGE has been used in the Encyclopedia of DNA Elements (ENCODE) project to detect TSS in several human cell lines [4], improve gene annotations [5] and to provide anchor points for epigenetic and other types of data [6].

Essential steps in the data processing of CAGE data include initial demultiplexing and read trimming steps, filtering, mapping reads to the genome and clustering of the mapped reads into peaks. The latter can be used to discover novel regulatory motifs and detect differential promoter usage among several samples.

To automate and standardize the analysis of CAGE data we created a web based workflow system called MOIRAI. Our goal was to create a simple system usable by both wet and dry scientists while at the same time providing an appropriate level of flexibility to meet project specific challenges. We include workflows to support the analysis of tagging-CAGE [2] and nano-CAGE [7] datasets.

## Implementation

Much like other workflow systems such as Galaxy [8], a MOIRAI workflow strings together several tools to process data in a stepwise fashion. Workflows can be created and modified using a built in graphical editor. The latter can be run as a stand alone application to prototype workflows before incorporating them into a web-based instance of MOIRAI.

Once fixed, users can run workflows by specifying input files and parameters via a simple web interface. A daemon in the background identifies pending jobs and executes them either locally or on a compute cluster. For each individual step in a workflow MOIRAI keeps track of the processing time, file sizes and potential error messages. All the results are written to a single directory containing a html page displaying the workflow used, detailed information about all programs/parameters used and links to the output files. In larger collaborations, it is beneficial to share the entire MOIRAI output as it contains both the data and the exact description of all processing steps. Several additional tools are included to simplify the installation and updating of analysis software, reference genomes and setting of in- and output directories per user.

### Installation

MOIRAI can be installed on UNIX based operating systems (tested on MacOSX, Ubuntu and Fedora) running Apache and PHP services. To make installation easy, we prepared an automatic script to set up MOIRAI on user’s personal computer. Using it, users will be able to run a default "Hello World" test workflow included in the download package after minutes of installation. Other CAGE workflows require the installation of analysis software, reference genomes and configuration of input and output directories. We provide the tools and scripts to automate these steps.

### Workflow overview

Each box in a MOIRAI workflow represents one process and an arrow between units describes a flow of the data. When a unit has multiple inputs, it will not be executed until all previous computations are completed. Furthermore, in the case of paired-end sequencing data, MOIRAI will verify that input files belong to the same sample by matching the file names. All units are colored to reflect three basic types of entities. Input files and reference databases are colored green, intermediate steps producing temporary files are gray and finally all outputs are shown in blue. For quality control purposes, we provide graphs embedded within the workflow to summarize the results at key steps. Similarly, thumbnails of windows looking similar to unix terminals allow users to check summary statistics and look at the most frequent sequences.

### Importing tools

To add flexibility, MOIRAI contains a standardized web-based mechanism to incorporate new command line tools and methods into the system. In brief, users can make an entry into an online table. Each entry requires the user to set the name of the program, the in- and output file formats, options and default parameters. It is possible to include the same program several times but with different parameter options to customize the resulting unit for particular types of data. Once incorporated these units can be used to create new workflows or modify existing ones.

Table 1 describes the necessary fields and entries to create a MOIRAI unit to convert SAM into BAM files using samtools [9]. In this case the unit does not accept any parameters and therefore only performs the conversion task. If incorporated into a workflow users will not be able to access other samtools options or functionality.

**Table 1:** Importing SAM to BAM functionality

**Format**	**Value**
Input	input=SAM
Output	output=BAM
Command line	samtools view -bSo [output] [input]
Parameters	NA

In contrast, Table 2 describes the necessary entries to incorporate the BWA mapper [10] into MOIRAI. Here the acceptable error rate is specified as a parameter and given a default value of 0.04. When a workflow incorporates this unit the default value is used but users have the option to modify it before starting their jobs. The mechanism described above can be used to give flexibility to pipelines while under development but reduce flexibility in a production environment.

**Table 2:** Importing BWA align functionality

**Format**	**Value**
Input	input=FASTQ reference=FASTA
Output	output=SAI
Command line	bwa aln -n [error-rate] [reference] [input] > [output]
Command parameter	error-rate=NUMBER
Default value	error-rate=0.04

### Available tools

MOIRAI comes with a basic set of tools required for CAGE processing. These include samtools [9], bwa [10] and bedtools [11]. In addition we use many standard UNIX commands, turned into units as described above, for many processing steps. Finally, we have developed tools specific for CAGE data processing including tools to demultiplex CAGE libraries, filter out rRNA and other unwanted reads, as well as a custom database (Table 3). These computation intensive tools are written in C and are parallelized for efficiency. We will briefly describe each of these tools in turn.

**Table 3:** Tools available in MOIRAI

**Tool**	**Description**
SplitByBarcode	Demultiplexing for CAGE
TagDust	Remove artificial sequences
rRNAdust	Remove rRNA sequence
SAMstat	Statistics of reads
Tome	Expression database
Graph	Draw PNG graphs from text table

CAGE tags from different libraries are tagged with barcode sequences and pooled into one sequencing lane to improve sequencing capacity and to enable accurate comparison between samples [12]. We wrote tools to demultiplexing these pooled tags into separate outputs according to barcode sequences for both single-end and paired-end CAGE. The MOIRAI workflow system will automatically recognize that demultiplexing units produce multiple files and will apply all downstream steps to all the files.

After demultiplexing we use two tools to remove unwanted sequences. TagDust [13] removes reads similar to primer dimers and other artifacts while rRNAdust removes reads matching ribosomal RNA sequences.

The tome database system is a simple implementation of a compressed sparse row data structure to store mapped CAGE reads from multiple experiments. The database can be queried to extract the number of CAGE reads within genomic boundaries given in bed6 format. The latter can be used to calculate the fraction of reads in each library mapping to promoters and to cluster biological samples.

SAMstat [14] displays mismatch, insertion/deletion error profiles, mapping rates and many other useful statistics of FASTQ/SAM/BAM sequence reads in HTML format. The report helps users to spot biases and problems in sequencing runs and protocols.

In addition we included several generic plotting tools to display the base distribution in intermediate sequence files and to list the most frequent sequences. These tools are extremely useful to spot and correct problems in complicated pipelines.

### Summary of results

Once a workflow is completed, MOIRAI summarizes the results and additional information on the run itself in a web page. The latter includes paragraphs describing each step including the version and parameters of the software used. By default all standard error and output messages are recorded per process and are stored in a separate html page. The run time and resulting file sizes of each step can be viewed by moving the mouse over the corresponding box in the workflow. Finally, the main output files are accessible by clicking on blue output boxes.

In addition to the results, we include a copy of the workflow template used for the computation itself. The workflow can be imported into another user’s account or MOIRAI instance to precisely reproduce all the results.

### Edit workflow

We included a workflow editor that can be accessed via the web-interface or as a stand alone application. The advantages of using the stand alone application include dragging and dropping data files directly from the desktop into the editor, offline editing of workflows as well as the ability to selectively execute only the recently added steps. The latter is desirable when debugging large workflows with time consuming initial processing steps. The mechanism for incorporating new tools and setting parameters is simple.

## Results

To demonstrate the use of MOIRAI we mapped, annotated and clustered eleven ENCODE CAGE libraries from the K562 cell line (Table 4). We will briefly describe each of the corresponding workflows in turn.

**Table 4:** ENCODE CAGE K562 Libraries

**Localization**	**Extract**	**Protocol**	**Replicate**
Polysome	longNonPolyA	nanoCAGE	
Chromatin	TotalRNA	nanoCAGE	
Nucleoplasm	TotalRNA	nanoCAGE	
Nucleolus	TotalRNA	nanoCAGE	
Nucleus	longPolyA	CAGE	biological
Nucleus	longPolyA	CAGE	biological
Cytosol	longPolyA	CAGE	biological
Cytosol	longPolyA	CAGE	biological
Cytosol	longPolyA	CAGE	
Whole cell	longPolyA	CAGE	biological
Whole cell	longPolyA	CAGE	biological

### CAGE mapping workflow

**Figure 1:**
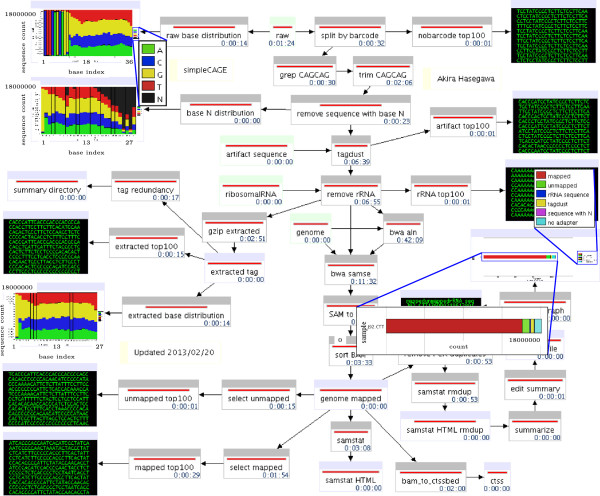
**A screenshot of the MOIRAI workflow for aligning CAGE sequences to a reference genome.** Each box represents one process and a direction of arrow shows flow of data. Computation starts from input units represented by green boxes. Gray boxes represent computational units where temporary files are deleted after workflow completes. Results are kept by redirecting them to file/directory units represented by blue boxes. Content of text/image file can be embedded and shown within a workflow for displaying final products or for checking quality of data production.

The mapping workflow is organized into three basic tasks: (a) raw reads are demultiplexed and trimmed, (b) artifacts and reads corresponding to ribosomal RNA are filtered out and (c) the actual mapping of the remaining reads. We placed quality control units on the left and right hand side of the workflow (Figure 1).

A multiplex sequence and a "CAGCAG" linker sequence at the beginning can be readily identified in the top left plot showing the base distribution of the raw reads. After demultiplexing and read trimming the base distribution is much more even (middle left plot). By default, we remove reads containing ambiguous bases (N). We can see from the second plot on the left that these low quality bases (shown in black) accumulate at the 3’ end of the reads which is typical for the Illumina sequencer [15].

Finally, a bar graph at the end of a workflow summarizes how many reads passed each stage. In the example shown we conclude that the CAGE reads are of high quality since only 2.4% of sequences were removed by filtering steps, and more than 90% of the filtered tags were successfully mapped.

Based on our experience, we consider a CAGE library to be of acceptable quality if fewer than 10% of the reads correspond to ribosomal RNA and if the mapping rate higher than 70%.

### Annotation workflow

**Figure 2:**
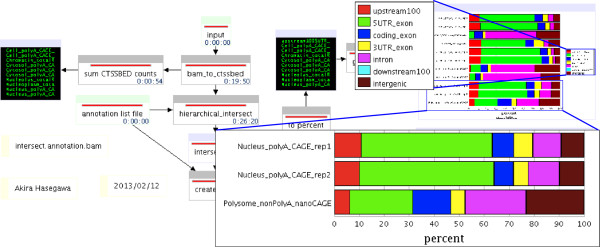
CAGE annotation based on Refseq.

The library annotation workflow takes mapped CAGE reads from multiple libraries and annotates them according to user specified rules (Figure 2). Here, we obtained gene models from UCSC [16] and used intersectBed to determine the fraction of CAGE reads at 100bp upstream, 5’ UTR exon, coding exon, 3’ UTR exon, intron, and 100bp downstream categories. We intersect the CAGE data with the annotation files hierarchically to make sure each CAGE read is only annotated to a single category. All remaining tags are categorized as intergenic. The results are summarized in a tab delimited table and shown as a graph in the workflow. While being designed with CAGE data in mind the same mechanism can be used to create any annotation pipeline based on any genome annotation.

In the case of the ENCODE CAGE libraries, it is evident that libraries obtained from nuclear sub-compartments have fewer reads mapped to promoters (100bp upstream and 5’UTR category) compared to the other libraries. As expected, the polyA+ libraries have high percentage of promoter regions compared to nuclear compartments as most measured RNA molecules are messenger RNAs.

### Expression analysis workflow

Finally, the expression analysis workflow groups mapped reads into peaks using a parametric clustering algorithm implemented in the program Paraclu [17]. In brief, this method reports genomic intervals containing many more CAGE reads than surrounding regions. These regions can be contained within each other giving rise to hierarchies of clusters. From this we select all clusters of length ≤ 200bp and with a stability greater than two. Normalized expression values for these peaks in multiple samples is extracted using the tome program.

**Figure 3:**
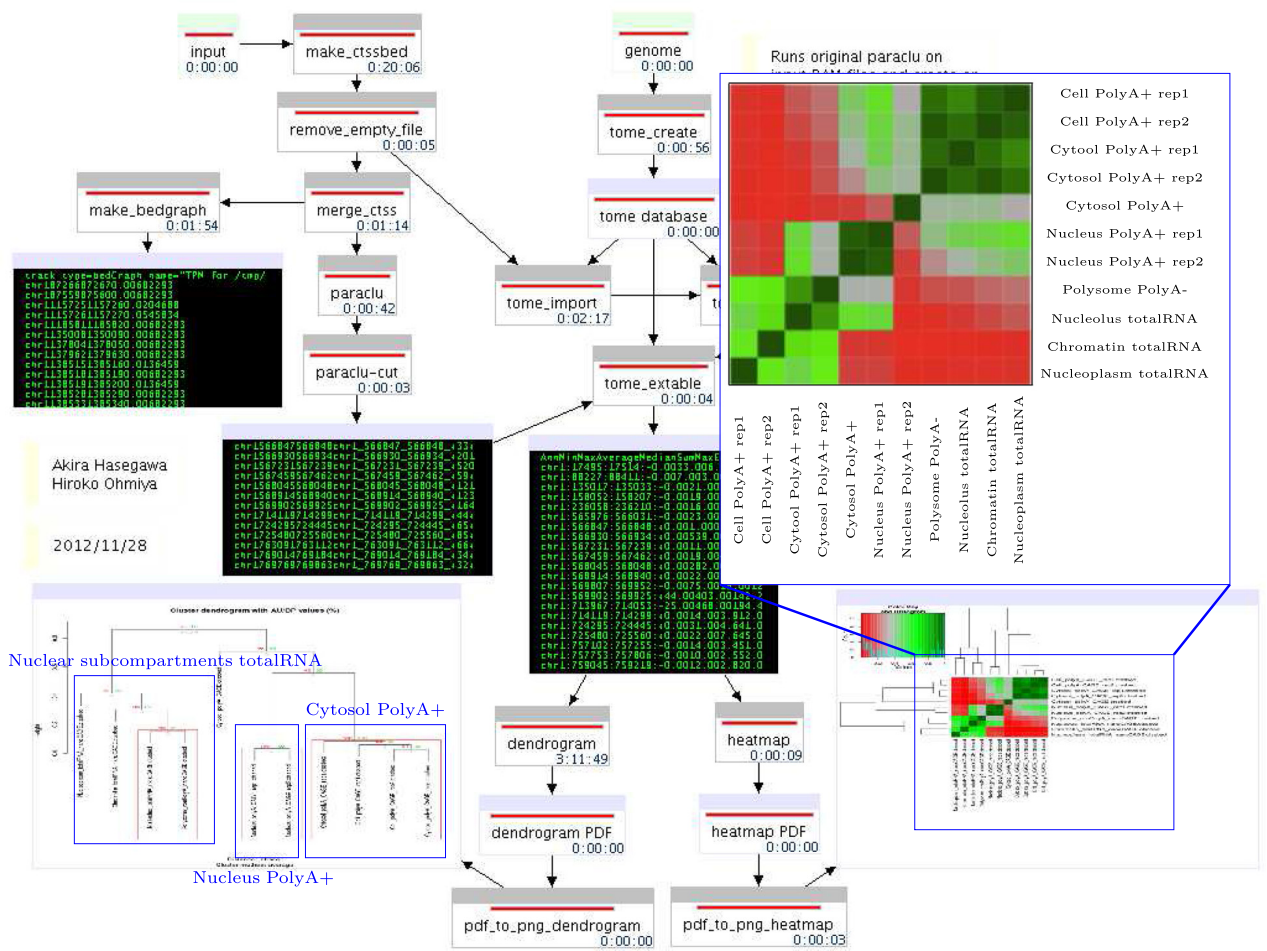
Hierarchal clustering of samples based on the expression of CAGE peaks.

The tome database itself is saved and can be used independently of the computed workflow. Based on the expression within the CAGE peaks the workflow calculated the Pearson’s correlation between samples and plots the results as a dendrogram and heat map (Figure 3).

The dendrogram graph is drawn by pvclust where probability values (p-values) are computed for each cluster with bootstrap resampling techniques [18]. If approximately unbiased (AU) p-value and bootstrap probability (BP) value are not necessary, bootstrap can be switched to computationally quicker hierarchical clustering (hclust). Biological replicas cluster together and libraries obtained from the whole cell and cytoplasmic fraction cluster closer together than nuclear libraries. One cytosolic polyA+ replica is clustered far away from other two replicate libraries.

The heatmap is another way of visualizing the overall similarity among samples. The graph shows two clusters of nuclear sub-compartments and cytoplasmic fractions with nuclear polyA+ replicates in the middle. It is interesting to note that nucleus polyA+ libraries appear to be similar to both nuclear and cytoplasmic libraries. Light green patterns appearing between all polyA+ samples indicates large number of mRNAs with polyA+ in nucleus are transported out to cytosol.

## Discussion

MOIRAI allows users of all skill levels to carry out the most essential steps in the processing and analyzing CAGE data. A key feature is the integration of analysis software and quality control programs in the same workflow. The latter is very useful in troubleshooting but also in getting a sense of computational bottlenecks and general flow of the data.

Finally, the output of MOIRAI combines result files with a html page showing the workflow used, all included programs, their versions and intermediate results. We believe this combination makes it very clear what was done to the data and facilitates reproducible research [19]. A copy of the workflow used is included in the results to make it easy to run exactly the same workflow on new data.

## Conclusions

MOIRAI is the right tool for processing and analyzing CAGE reads. It is simple to use, provides flexibility to adjust analysis workflows/pipelines when needed while being capable of dealing with large amounts of NGS data.

Furthermore, we plan to expand workflows to downstream analysis including large scale data integration.

While we have focussed here on CAGE data, the inherent flexibility of MOIRAI makes it possible to generate workflows to process RNAseq, Chip-Seq and other types of data.

## Availability and requirements

**Project name:** MOIRAI

**Project home page:**http://sourceforge.net/projects/moirai/

**Operating systems:** Unix/Linux or Mac

**Programming language:** java, perl, php, c, bash

**Other requirements:** R, sqlite3, and Java SE (Standard Edition).

**License:** GNU General Public License version 3.0 (GPLv3)

**Others:** The MOIRAI package is also available from the fantom web resource ( http://fantom.gsc.riken.jp/software/).

## Competing interests

The authors declare that they have no competing interests.

## Authors’ contributions

AH created MOIRAI. AH, TL contributed analysis software. PC, CD, YH and TL supervised the work. All authors read and approved the final manuscript.
